# The Hepatoprotective Effect of Vitamin A against Gasoline Vapor Toxicity in Rats

**DOI:** 10.4021/gr2009.06.1297

**Published:** 2009-05-20

**Authors:** Friday E. Uboh, Itemobong S. Ekaidem, Patrick E. Ebong, Ime B. Umoh

**Affiliations:** a Department of Biochemistry, Faculty of Basic Medical Science, College of Medical Sciences, University of Calabar - Calabar, Nigeria; b Department of Chemical Pathology, University of Uyo – Uyo, Nigeria

**Keywords:** Hepatoprotective, Gasoline vapours, Retinol, Aminotransferases, Bilirubin

## Abstract

**Background:**

Changes in the activities of plasma alanine amino transferase (ALT), aspartate amino transferase (AST), gamma glutamyl transferase (GGT), and alkaline phosphatase (ALP) are used to assess the functional state of the liver. Significant increase in the activities of these enzymes commonly indicates the hepatotoxicity of chemical agent(s) in the body. Exposure of male and female rats to 17.8 cm^3^h^-1^m^-3^ of Premium Motor Spirit (PMS) blend unleaded gasoline (UG) vapors for 6 hr/day, 5 days/week for 20 weeks have been observed to cause hepatotoxicity. In this study, the potential hepatoprotective effect of vitamin A (retinol) against gasoline vapours-induced toxicity was investigated in male and female rats.

**Methods:**

Retinol (400 IU/kg/day) was orally administered to the test rats concomitant with the gasoline vapor exposure in the last two weeks of the experiment.

**Results:**

The results obtained from this study showed that exposure to gasoline vapors caused significant increase (P < 0.05) in the activities of serum ALT, AST, ALP, GGT and bilirubin in both male and female rats. The treatment of the male and female test rats with vitamin A produced a significant decrease (P < 0.05) in the activities of these parameters, compared with the test rats without treatment; but insignificant increase(P ≥ 0.05), compared with the control.

**Conclusions:**

The result of this study demonstrates the beneficial effects of retinol, at prophylactic dosage, against gasoline vapours hepatotoxicity in male and female rats, thereby suggesting that retinol may be used to prevent hepatotoxicity in individuals frequently exposed to gasoline vapours.

## Introduction

Gasoline, one of the fractionated products of crude oil, is widely used as fuels for automobiles and some electricity generating machines. Gasoline is known to be a very volatile liquid, with several organic and inorganic constituents. Gasoline vapors may be derived from direct evaporation of liquid gasoline. These vapors, which are ubiquitous in the environment and constitute some components of petroleum pollutants in the air, are of great environmental and human health concern. Exposures to these pollutants are common in the refineries, oil fields, refueling stations, petrochemical industries, motor mechanical workshops, and traffic-congested areas. However, the population at greater risk of frequent exposure includes those occupationally exposed. It has been reported that the oil drillers, refinery workers, petrochemical workers, refuel station attendants and motor mechanics suffer the greater risk of chronic exposures to petroleum pollutants [1-3].

Literature report also showed that more saturated than unsaturated aromatic hydrocarbons are found in human and animal blood after inhalation exposure to equal concentrations [4]. In animals, gasoline vapors have been shown to be toxic to many tissues. In our previous studies, it was reported that gasoline vapors induced proatherogenic changes in serum lipid profile and signs of hepatic oxidative stress [5-7], haematotoxicity [8, 9], reproductive toxicity [10] and nephrotoxicity [11], in male and female rats. The molecular mechanisms through which gasoline vapors’ constituents and other chemical agents express their toxicity effects may vary. For instance, the molecular mechanism that may be responsible for the toxicity of cadmium has been reported to involve oxidative stress which disturbs the antioxidant defense system and produces reactive oxygen species (ROS), including hydrogen peroxide, superoxide and hydroxyl radical [12].

In the recent times, the major concern of the environmental and biochemical toxicologists has been how to devise measures that can abate or reverse the adverse effects associated with exposure to environmental pollutants. Since the toxicity effect associated with exposure to gasoline vapors’ constituents may be an indication of tissue, or tissue components – reactive metabolite species interactions in the body; it is believed that the presence of antioxidants may ameliorate the toxicity effect. Some antioxidants are naturally present in the body, while others have to be provided as micronutrients. Some vitamins are known to play an important role in ameliorating the toxicity effects of reactive species generated by chemical agents in the biological systems. Vitamins A and E are among the antioxidants vitamins that have attracted the attention of biochemical and toxicological researchers in the recent times. Vitamin A is reported to enhance a marked reduction in CCl-induced hepatic damage in mice [13]. We have also observed and reported that administration of vitamin A produced a significant regain in weight loss, growth depression and haematotoxicity resulting from exposure to gasoline vapors in male and female rats [14]. It has been documented that the primary antioxidant role of vitamin A is to scavenge singlet oxygen; the singlet oxygen reacts with lipids to form lipid hydroperoxides, and the removal of singlet oxygen prevents lipid peroxidation [15]. Also, hepatoprotective effect of β-carotene against cadmium toxicity in rats has been reported [Bashandy and Alhazza, 2008]. Hence, among the various biochemical functions of vitamin A, its antioxidative and protective role have attracted more investigations in the recent times [16-18]. Retinol and the related compounds have been reported to possess apparent ability to interfere with some chemical reactions that may potentiate carcinogenesis [19].

Administration of retinol and other retinoids to animals is also reported to delay arrest and even reverse progression of premalignant cells and malignant characteristics. Vitamin A, a member of retinoid family, is obtained from β-carotene. It exists in several chemical forms, such as retinol, retinoic acid and retinal. Interconversions between these chemical forms readily occur in the body. Vitamin A is also present as a retinyl ester in the tissues of animals. Among the various biochemical functions of the vitamin A, its antioxidative and protective role has attracted more investigations in the recent times [16, 17]. Retinol and the related compounds are reported to possess apparent ability to interfere with carcinogenesis. This study was designed to assess the comparative protective potential of vitamin A against gasoline vapors-induced hepatotoxicity in male and female rats.

## Materials and Methods

### Experimental animals

Forty-eight matured Wistar albino rats (twenty-four females and twenty-four males) weighing 158.5 ± 12.3g were obtained from the animal house of the College of Medical Sciences, University of Calabar, Calabar – Nigeria, and used for this study. The rats were divided into six groups with eight rats each, as follows: Group I (Mc): Male control group, without exposure to gasoline vapours; Group II (Mt): Male test group, exposed to gasoline vapors only; Group III (MvitA): Male test group concomitantly administered with vitamin A daily for the last two weeks of the exposure; Group IV (Fc): Female control group, without exposure to gasoline vapors; Group V (Ft): Female test group, exposed to gasoline vapors only; Group VI (FvitA): Female test group concomitantly treated with vitamin A daily for the last two weeks of the exposure.

The rats were acclimatized in the experimental animal house for one week before the commencement of the experiment. The animals, housed in stainless steel cages under standard conditions (ambient temperature, 28 ± 2 °C and humidity,46%, with a 12 hr light/dark cycle), were fed with the normal rat pellets. All the rats in both test and control groups were allowed free access to food and water *ad libitum*, throughout the experimental period. All the animal experiments were carried out in accordance with the guidelines of the Institution’s Animal Ethical Committee.

### Exposure to gasoline vapors

The animals in the test groups were wholly exposed to Premium Motor Spirit (PMS) blend unleaded gasoline (UG) vapours in a glass exposure chambers (1.5 m x 0.9 m x 2.1 m). The PMS blend liquid UG was obtained from Mobil refueling station, Marian Road, Calabar – Nigeria. The test animals were exposed to 17.8 cm^3^h^-1^m^-3^ (target concentration) of wholly vaporized PMS blend UG for 6 hr/day, 5 days/week, for 20 weeks. Exposure conditions were chosen to reproduce those used in our previous studies [5, 7, 8, 11]. Exposures were routinely conducted from 9.00 am to 3.00 pm on week days, including holidays to mimic workplace exposure. The chamber design, exposure generation system, and monitoring system were the same as those previously described [5, 7, 8, 11] with chamber concentrations of the UG determined daily. The average daily chamber concentrations of UG during exposure periods were 17.8 ± 2.6 cm^3^ (about 85 .4 percent of target concentration). At the end of the experimental period, the animals were sedated with chloroform and dissected for collection of blood specimen.

### Treatment of the rats with vitamin A

After eighteen weeks of pre-inhalation exposure to the gasoline vapors, the rats in Groups III and VI were respectively administered, once daily, with 400 IU/kg of vitamin A (retinol), i.e., at normal prophylactic dose, concomitantly with exposure to gasoline vapors for the remaining two weeks. Administration of the vitamins was done by oral gavaging using intragastric syringe after solubilizing the vitamin with Goya Olive oil, as the vehicle.

### Collection and preparation of blood specimen for analyses

Blood samples were collected by cardiac puncture into plain screw-cap sample bottles. The blood samples collected were allowed to clot, and the serum extracted with Pasteur pipette after spinning with MSE model (England) table-top centrifuge at 2000 rpm for 5 minutes. The serum collected was used for biochemical analyses. All biochemical analyses were carried out within 24 hours of serum separation.

### Biochemical analyses

Biochemical analyses carried out included measurement of the concentration of alanine transaminase (ALT), aspartate transminase (AST), gamma-glutamyltransferase (GGT), alkaline phosphatase (ALP), and bilirubin in the serum. The measurements of the concentrations of these biochemical parameters were done by spectrophotometric determination of their absorbances, using analytical grade laboratory reagent kits. The laboratory reagent kits from Biosystems Laboratories (S. A. Costa Brava, Barcelonia, Spain) were used to assess the concentration of ALT, AST and ALP in the serum. While reagent kits from Randox Laboratories (United Kingdom) were used to assess the concentration of GGT, and bilirubin in the serum. All absorbance readings were taken with DREL3000 HACH model spectrophotometer.

### Statistical analyses

The results were analyzed by one-way analysis of variance (ANOVA) followed by Student’s t-test to evaluate the significance of the difference between the mean value of the measured parameters in the respective test and the control groups. A significant change was accepted at P < 0.05.

## Results

**Figure 1:**
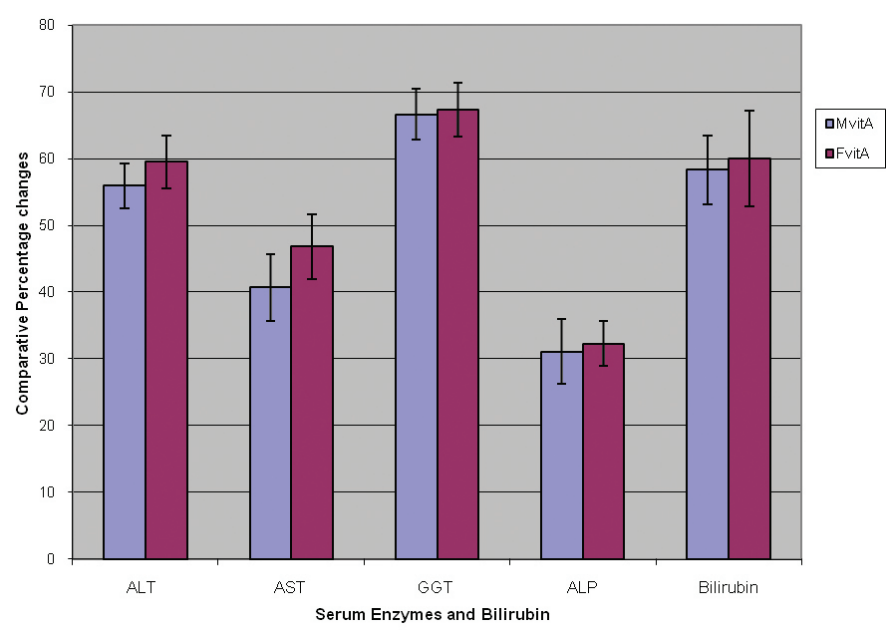
Effect of vitamin A on comparative percentage changes in the activities of some serum enzymes and bilirubin in male and female rats exposed to gasoline vapors.

The results of this study are shown in Table 1 and Figure 1. These results showed that the activities of ALT, AST, GGT and ALP obtained for the male and female experimental test rats, exposed to gasoline vapor only (i.e, Mt and Ft ), were increased significantly (P < 0.05) compared, respectively, with the activities obtained for the respective rats in the control group ( i.e., Mc and Fc). The results indicated that exposure to gasoline vapors increased the activities of ALT, AST, GGT and ALP by 62.4 ± 5.3, 48.3 ± 4.8, 69.0 ± 6.3 and 32.7 ± 3.9 percents respectively in males, and 65.7 ± 4.2, 51.5 ± 3.8, 71.5 ± 5.6 and 33.2 ± 5.2 percents, respectively, in female rats. From these results, it is observed that the percentage increase in the activities of these enzymes in female rats was relatively higher compared, respectively, to the percentage increase obtained for the male rats.

**Table 1:** Effect of vitamin A on the activities of some serum Enzymes and bilirubin in male and female rats exposed to gasoline vapours

GROUP	ALT (U/L)	AST (U/L)	GGT (U/L)	ALP (U/L)	BILIRUBIN (µmol/L)
I (Mc)	8.85 ± 2.0	19.82 ± 1.70	26.30 ± 3.44	263.15 ± 12.84	2.05 ± 0.4
II (Mt)	23.85 ± 1.01*	38.34 ± 1.95*	84.81 ± 9.95*	390.79 ± 14.33*	4.99 ± 0.28*
III (Mvit A)	10.39 ± 1.22^+ ∧^	22.72 ± 2.50^+ ∧^	28.33 ± 5.51^+ ∧^	269.41 ± 8.71^+ ∧^	2.08 ± 0.28^+ ∧^
IV (Fc)	8.35 ± 1.64	17.63 ± 2.06	17.97 ± 1.49	250.24 ± 8.73	2.17 ± 0.40
V (Ft)	24.31 ± 0.77**	36.38 ± 1.95**	62.96 ± 6.59**	374.65 ± 18.01**	5.50 ± 0.61**
VI (Fvit A)	9.85 ± 1.58^++ #^	19.37 ± 0.54^++ #^	20.59 ± 6.14^++ #^	253.61 ± 9.53^++ #^	2.20 ± 0.42^++ #^

Values are presented as mean ± SEM, n = 8, *P < 0.05 compared with group I (control); ^+^P < 0.05 compared with group II; ∧P > 0.05 compared with group I; **P < 0.05 compared with group IV (control). ^++^P < 0.05 compared with group V; ^#^P > 0.05 compared with group IV. Mc = male control; Mt = male test, exposed to gasoline vapors only; MvitA = male test treated with vitamin A; Fc = female control; Ft = female test, exposed to gasoline vapors only; FvitA = female test treated with vitamin A.

The activities of ALT, AST, GGT and ALP in male and female rats treated with vitamin A were significantly lower (P < 0.05) compared respectively to the activities obtained for male and female rats exposed to gasoline vapors only (Table 1). However, the percentage decrease in the activities of these enzymes in female rats treated with vitamin A was observed to be insignificantly (P ≥ 0.05) higher than the respective percentage decrease obtained for male rats treated with vitamin A (Fig. 1).

Moreover, exposure to gasoline vapors also resulted in a significant increase (P<0.05 ) in the level of bilirubin by 58.9 ± 5.7 and 60.5 ± 6.3 percents, respectively, in male and female rats compared to the control. It was also observed that the level of bilirubin in male and female rats treated with vitamin A was significantly (P < 0.05 ) lower than that of rats exposed to gasoline vapors only, and that the percentage decrease obtained for female rats was also insignificantly (P ≥ 0.05 ) higher than that obtained for the male rats (Fig. 1). The results obtained from this study indicated that vitamin A may enhance recovery from hepatotoxicity associated with exposure to gasoline vapors in male and female rats, with the females benefiting relatively than the males.

## Discussion

Exposure to gasoline vapors causes a wide spectrum of toxicological effects, as well as biochemical dysfunctions that constitute serious health hazards to humanity. Increased lipid peroxidation and oxidative stress in hepatocytes of male and female rats have been reported to be associated with exposure to gasoline vapors [6]. This report indicates that gasoline vapors’ constituents participate in oxidative reactions associated with generation of some reactive species which interact with membrane lipids of hepatocytes to produce lipid peroxides, as reported for cadmium [20]. Such reactive oxygen species as hydroxyl and superoxide radicals are known to provoke severe cellular alterations resulting in cell damage or death, due to their high reactivity. These species attack such important cell constituents as proteins, lipids and nucleic acids, and the lipid peroxides that accumulate due to lipid peroxidation are known to be very harmful to cells and tissues [21]. The liver has been shown to be one of the target organs of gasoline vapors toxicity [8]. The relationship between the hepatic oxidative damage and increase in the activities of such serum enzymes as ALT, AST, ALP and GGT has been well documented [7, 8, 22, 23]. Exposure to gasoline vapors stimulates cellular malondialdehyde (MDA), a product of lipid peroxidation [6], which affect the permeability barrier of the plasma membrane. The result of this study gives an indication that the hydrocarbons and other chemical constituents of the gasoline vapors are likely metabolized in the liver, among others, to reactive species which interact with the tissues to cause lipid peroxidation, thereby exhibiting their toxic or hazardous effects. The observed increase in the activities of plasma ALT, AST and ALP is likely to be due to lipid peroxidation of biomembranes which causes leakage of cellular components [24]. Therefore, it is likely that the increase in the liver enzymes reported in this present study may be due to the accumulation of gasoline vapors’ constituents and their reactive metabolites in hepatic tissues which enhances formation of lipid peroxidation.

A significant increase in the level of serum bilirubin was also observed in male and female rats exposed to gasoline vapors when compared with control rats. Generally, increase in plasma or serum bilirubin level may be an indication of either or all of the following; excessive production of bilirubin as in haemolytic anaemia, reduced hepatic uptake as in the liver subjected to potential damage from an enormous array of chemical agents, impaired bilirubin conjugation, decreased hepatocellular excretion, and impaired intrahepatic and extrahepatic bile flow [25, 26]. Although the specific mechanism(s) leading to the elevation of the total serum bilirubin level, reported in this study, is not very clear, the result from enzyme studies give an indication that the gasoline vapors’ constituents might have interacted with the liver tissues to raise the total serum bilirubin level through one or more of the mechanisms earlier outlined by Crawford [26]. This observation supports our earlier and present reports that frequent exposure to gasoline vapors may induce hepatotoxicity, with diverse clinical complications, and that the hepatotoxic effects associated with exposure to gasoline vapours may be diverse. The results of this present study agrees with our previous report that the levels of serum ALT, AST, ALP, GGT and total bilirubin, as well as relative liver weight in male and female rats were increased following frequent exposure to gasoline vapors.

In this present experiment, administration of vitamin A reduced gasoline vapours-induced elevation of ALT, AST, ALP, GGT and bilirubin level in the serum, indicating that vitamin A possesses hepatoprotective property against gasoline vapors toxicity in male and female rats. The result obtained from this investigation correlates the report of Bashandy and Alhazza [22], that β-carotene protects the liver against cadmium toxicity in rats; this report records that pretreatment with β-carotene reduced cadmium-induced elevation of ALT, AST and ALP in rats. β-carotene, the precursor of vitamin A, has been reported to suppress lipid peroxidation elevation in rats by quenching the activities of free radicals and prevent them from inducing oxidative stress [27, 28]. According to McNulty *et al* [29], carotenoids quench singlet oxygen primarily by physical mechanism, in which excess energy of the singlet oxygen is transferred to carotenoids and then they relax into ground state, as a result carotenoids offer to protect against further oxygen radical and lipid peroxidation. Vitamin A, like β-carotene, is believed to improve the activities of antioxidative enzymes following oxidative stress; and being a lipophilic molecule, it is likely to exert its action in such hydrophobic environment as the lipid core of membranes. Prevention of reactive metabolites formation or rapid scavenging of the generated reactive species by the antioxidants may be useful in preventing the toxicity effects of different reactive metabolites. For example, Peshlon and Hesse [30], Maellaro *et al* [31], and Sheweita *et al* [32] reported that antioxidants have proved to be effective in protecting the liver against carbon tetrachloride-induced hepatotoxicity.

With the gasoline vapours-induced toxicity protective role of vitamin A reported in this present work, it may be postulated that vitamin A can act as an antioxidant, preventing or reversing the toxic effects of the various reactive metabolites responsible for the hepatotoxicity, observed to be associated with exposure to gasoline vapors. The result of this work also indicated that the beneficial effect of vitamin A against gasoline vapors-induced hepatotoxicity was relatively higher in female than male rats. The specific mechanism of the observed relative sex-dependent hepatoprotective beneficial effect of the vitamin is a subject for further investigation. However, it may be concluded from the result of this study that vitamin A may be used to enhance recovery from, or prevent hepatotoxicity associated with exposure to gasoline vapors. Hence, this vitamin may be recommended at prophylactic dosages to those who are routinely occupationally exposed to gasoline vapors.
